# Syndrome Valentino From a De Novo Aetiology – Acute Pancreatitis

**DOI:** 10.7759/cureus.21360

**Published:** 2022-01-18

**Authors:** Balasubramanian Arumugam, Bhanumati Giridharan, Prabhakar R, Shanmugasundaram P.N.

**Affiliations:** 1 General Surgery, Employees State Insurance Corporation (ESIC) Medical College & Post Graduate Institutes for Medical Sciences & Research (PGIMSR), Chennai, IND

**Keywords:** anterior pararenal space, duodenal perforation, valentino syndrome, acute pancreatitis, acute appendicitis

## Abstract

Valentino syndrome is one of the rare classical presentations of duodenal perforation, wherein the leaked contents collect at the right lower quadrant of the abdomen causing local peritonitis and mimicking appendicitis. Here we present a case profile of a 28-year-old gentleman, who presented with right lower quadrant abdominal pain and mass, which was clinically diagnosed as acute appendicular inflammatory mass. Later with laboratory reports and radiological imaging, he was confirmed to have acute pancreatitis, and actually the peripancreatic fluid collection has tracked down into the right iliac fossa and pelvis to present similar to Valentino syndrome. This article is reported to highlight acute pancreatitis as a cause of Valentino syndrome.

## Introduction

The Valentino syndrome or Valentino’s appendix was originally named after an American actor, Rudolph Valentine (1895-1926). He had an appendectomy for his acute appendicitis-like presentation but later died due to peritonitis and multiorgan failure. His autopsy revealed a perforated gastric ulcer and the leaked contents were tracking through the paracolic gutters into the right iliac fossa causing peritoneal irritation in that area, mimicking appendicitis [1]. This eponym is used since then to emphasize the rare presentation of perforated peptic ulcer disease as appendicitis. In this article, we report a case of acute pancreatitis presenting similarly to Valentino syndrome.

## Case presentation

**Figure 1:**
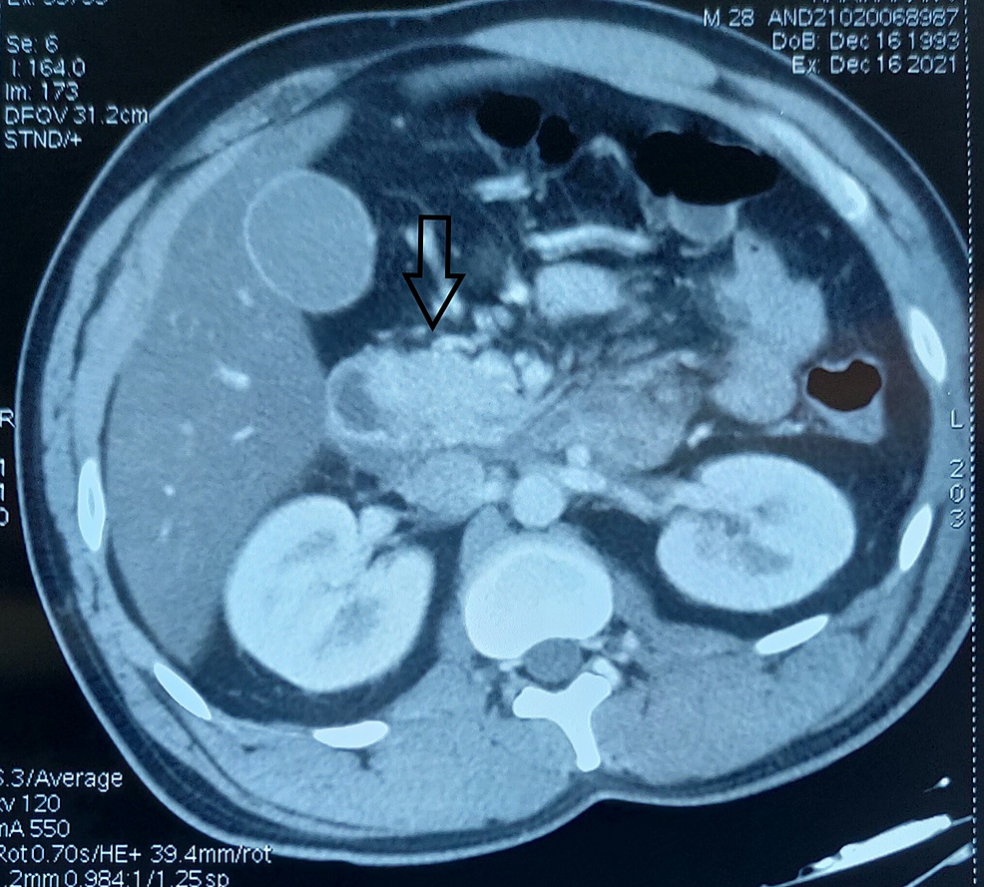
Acute edematous and bulky inferior part of the head and uncinate process of the pancreas (arrow).

**Figure 2:**
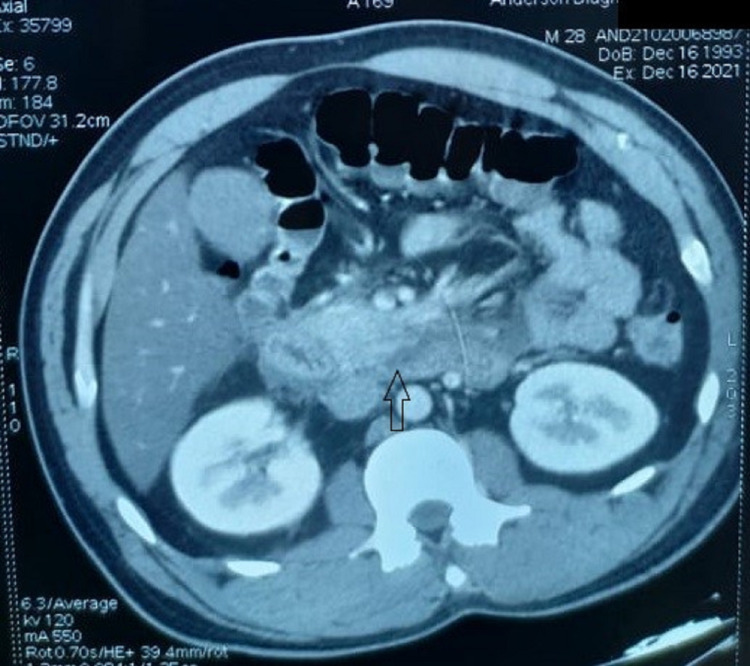
Acute edematous and bulky inferior part of the head and uncinate process of the pancreas with peripancreatic fluid collection (arrow) noted in infra pancreatic region, pancreaticoduodenal groove and precaval region.

**Figure 3:**
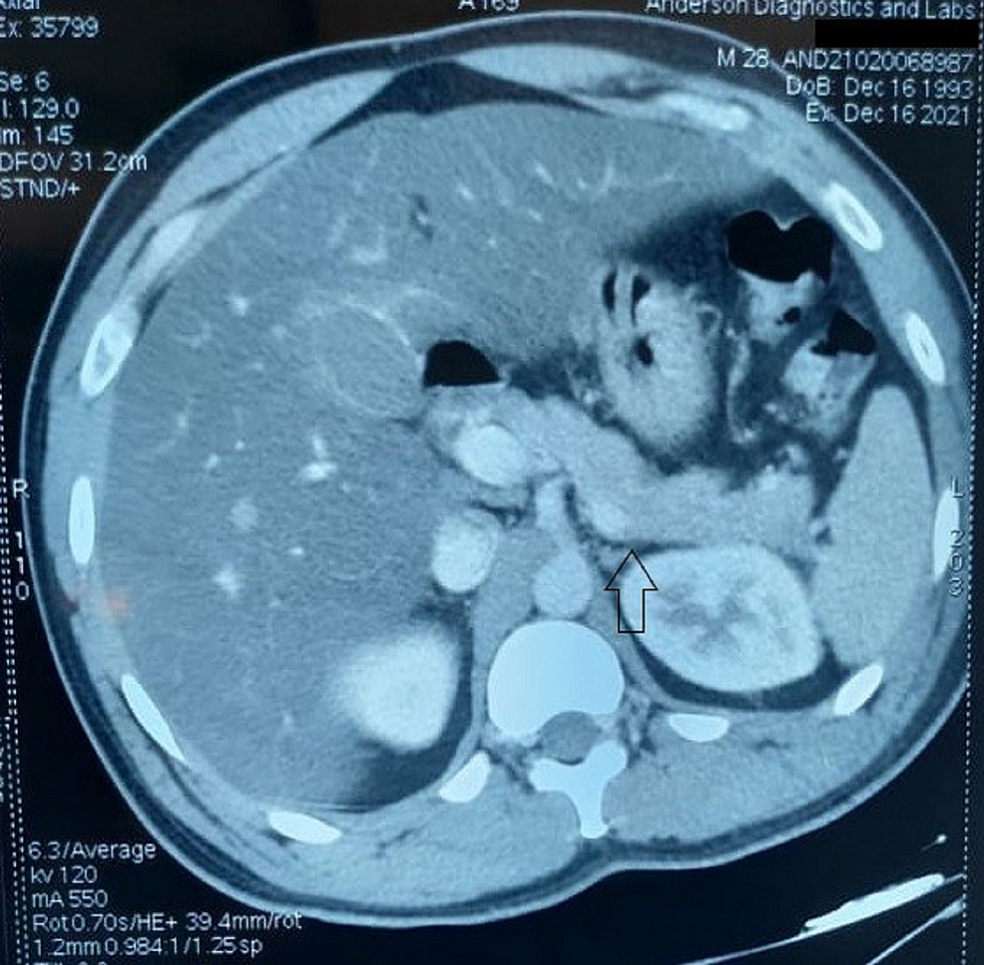
CT images showing the entire pancreas with a normal-appearing body and tail of the pancreas (up arrow).

**Figure 4:**
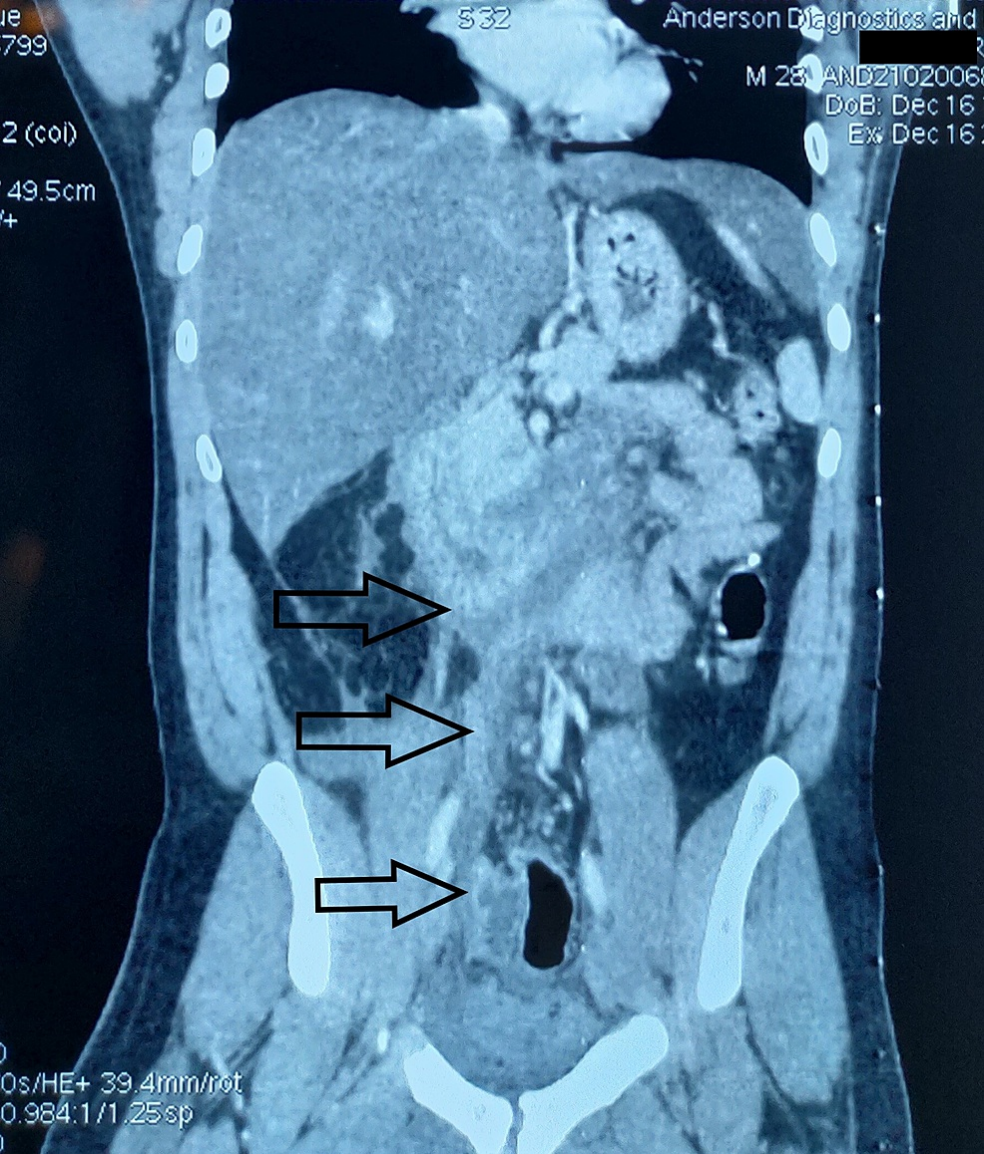
The peripancreatic fluid (arrows) extends into the right side anterior pararenal and paracolic gutters and then eventually into the right lateral pelvic wall and presacral region.

A 28-year-old gentleman presented in our surgical casualty with a four-day history of acute onset diffuse abdominal pain, which was now localized at the right lower quadrant of the abdomen. He had one episode of fever initially and had nausea with bilious vomiting for the same duration. He occasionally consumes alcohol and his last drink was one year ago. He did not have any relevant past medical and surgical history. On clinical examination, he was found to be afebrile, dehydrated, and had tachycardia (Pulse rate 140/min). The examination of the abdomen proper revealed a mild uniformly distended abdomen with right iliac fossa fullness and a localized tenderness over it. He was admitted with the provisional clinical diagnosis of appendicular inflammatory mass. His urgent ultrasonogram abdomen reported probe tenderness over the right iliac fossa region and could not document any bowel or visceral pathology due to excess gas shadows. The basic blood investigations suggested an inflammatory or infective pathology with total white cell counts of 13,270/cu mm (Neutrophils 78%) and C-reactive protein value of 252. The panel of blood tests also included an elevated serum amylase (177 U/L) and lipase (663 U/L), which prompted for an emergency contrast-enhanced computed tomography (CECT) of the abdomen and pelvis. The CECT abdomen gave an entirely different diagnosis of acute pancreatitis with a severity score of 6/10 (Figures 1-3). The detailed report revealed an edematous and bulky inferior part of the head and uncinate process of pancreas with the acute peripancreatic fluid collection noted in infra pancreatic region, pancreaticoduodenal groove and precaval region. This fluid collection was extending into the right anterior pararenal space, right paracolic gutter, right lateral pelvic wall and presacral region (Figure 4).

The imaging was vital in making the treatment decision in this patient, who would have otherwise had a laparotomy for the suspicion of the appendicular abscess. The patient was managed successfully with the conservative line of management with appropriate fluid hydration, analgesics and bowel rest. His cause of pancreatitis was found to be high levels of serum triglyceridemia in the next day fasting lipid profile (660 mg/dl) and then he was put on lipid-lowering agents with a low-fat diet plan. The patient was discharged after a week and is on regular follow-up.

## Discussion

Valentino syndrome was originally described as an eponym to impart caution to our surgical fraternity in handling cases of appendicitis-like clinical presentations [1]. The literature available so far accounts the peptic ulcer disease with gastric and duodenal perforation as the sole cause of this rare presentation [2,3]. Both intraperitoneal and retroperitoneal perforation can lead to Valentino’s appendix but the latter will be more difficult to diagnose clinically and hence more feared.

Acute pancreatitis with Valentino’s appendix-like presentation is probably the first time reported in the literature. Pancreas shares its neighbourhood with duodenum and stomach in the lesser sac and hence it can also present with similar findings in the presence of sufficient peripancreatic fluid collection. To understand the pathophysiology behind the Valentino’s appendix presentation, we require the knowledge of various retroperitoneal spaces and their communications with each other. To brief a little about it, retroperitoneal space is that space between parietal peritoneum and posterior fascia transversalis. It is divided into three compartments, that is, lateral, median (vascular) and posterior (muscular) compartments [4]. The lateral compartment is further divided into three spaces namely the anterior pararenal, perirenal and posterior pararenal. The relevant space for Valentino’s appendix presentation is the anterior pararenal space which is bilateral and communicates with each other. It contains the duodenum, pancreas, ascending colon and descending colon. It has free communication within this wide space and follows the spaces along the mesentery and paracolic gutters up to the pelvis. Thus any fluid collection in this space of anterior pararenal area can potentially track along the mesenteries and paracolic gutters into the pelvis mimicking an appendicitis or a pelvic abscess.

The presentation in our case was typical of a Valentino’s appendix and he even had a right iliac fossa fullness and rebound tenderness due to the local peritoneal collection and irritation. The presentation was acute with a clinical picture pushing for an emergency laparotomy in view of suspected appendicular perforation or abscess. Only with a detailed panel of blood investigations for acute abdomen, CT imaging and clinical follow-up, we were able to make the right diagnosis and management plan for him.

## Conclusions

Acute pancreatitis can also present as appendicitis and will be even more difficult to identify with clinical acumen alone. Awareness and a systematic approach of ruling out the other possible causes of a presentation will be helpful in identifying and treating these rare conditions. The eponym Valentino’s appendix is worth to be remembered and passed on to the upcoming surgical generations for the plight it can save us from.
